# Non-Muscle Myosin II Isoforms Have Different Functions in Matrix Rearrangement by MDA-MB-231 Cells

**DOI:** 10.1371/journal.pone.0131920

**Published:** 2015-07-02

**Authors:** Bridget Hindman, Zoe Goeckeler, Kostas Sierros, Robert Wysolmerski

**Affiliations:** 1 Mary Babb Randolph Cancer Center, West Virginia University, Robert C. Byrd Health Sciences Center, Morgantown, West Virginia, United States of America; 2 Center for Cardiovascular and Respiratory Diseases, West Virginia University, Robert C. Byrd Health Sciences Center, Morgantown, West Virginia, United States of America; 3 Mechanical and Aerospace Engineering, Statler College of Engineering and Mineral Resources, West Virginia University, Morgantown, West Virginia, United States of America; University of California, San Diego, UNITED STATES

## Abstract

The role of a stiffening extra-cellular matrix (ECM) in cancer progression is documented but poorly understood. Here we use a conditioning protocol to test the role of nonmuscle myosin II isoforms in cell mediated ECM arrangement using collagen constructs seeded with breast cancer cells expressing shRNA targeted to either the IIA or IIB heavy chain isoform. While there are several methods available to measure changes in the biophysical characteristics of the ECM, we wanted to use a method which allows for the measurement of global stiffness changes as well as a dynamic response from the sample over time. The conditioning protocol used allows the direct measurement of ECM stiffness. Using various treatments, it is possible to determine the contribution of various construct and cellular components to the overall construct stiffness. Using this assay, we show that both the IIA and IIB isoforms are necessary for efficient matrix remodeling by MDA-MB-231 breast cancer cells, as loss of either isoform changes the stiffness of the collagen constructs as measured using our conditioning protocol. Constructs containing only collagen had an elastic modulus of 0.40 Pascals (Pa), parental MDA-MB-231 constructs had an elastic modulus of 9.22 Pa, while IIA and IIB KD constructs had moduli of 3.42 and 7.20 Pa, respectively. We also calculated the cell and matrix contributions to the overall sample elastic modulus. Loss of either myosin isoform resulted in decreased cell stiffness, as well as a decrease in the stiffness of the cell-altered collagen matrices. While the total construct modulus for the IIB KD cells was lower than that of the parental cells, the IIB KD cell-altered matrices actually had a higher elastic modulus than the parental cell-altered matrices (4.73 versus 4.38 Pa). These results indicate that the IIA and IIB heavy chains play distinct and non-redundant roles in matrix remodeling.

## Introduction

Breast cancer is a widespread disease that remains a leading cause of death in the US, despite public education and research initiatives in recent years. With 232,340 new cases of invasive disease estimated in 2013, and 39,620 expected deaths, breast cancer is the second leading cause of cancer related deaths in women [1]. An initial sign of breast cancer is the presence of a palpable lump in the breast [2]. This lump, or stiffening of the breast tissue, corresponds to up to a ten-fold increase in the rigidity of the extracellular matrix (ECM) of the tissue [3]. Changes to cell and/or tissue mechanics, such as the increased rigidity of the breast during cancer tumorigenesis, may have an influence on cell signaling, proliferation, invasion and migration [2, 4–6], and can therefore have a vast impact on how cancer is diagnosed and treated.

Tissues maintain a balance of overall stiffness by a phenomenon known as mechanoreciprocity. This involves a feedback loop between the cells and their surrounding matrix to maintain a particular rigidity [2, 7, 8]. In some diseases, including many solid cancers, this homeostasis is lost and promotes disease progression [2, 9]. This loss of homeostasis can be the result of changes in ECM content and cross-linking [3, 10], as well as the increased cell pressure caused by the high cell density within a growing tumor [4, 11]. In fact, these two facets of tissue stiffness can feed into each other. Tumor cells excrete factors that activate stromal cells, including fibroblasts, inducing them to deposit ECM components and secrete crosslinking factors such as lysyl oxidase. The resultant increased matrix rigidity in turn stimulates cell proliferation, which increases tumor cell density and pressure [2, 4, 5, 7, 11]. During the latter stages of disease progression, ECM stiffness and reorganization influences cancer invasion and metastasis [2, 4, 6, 10, 12–14]. Breaking the link between increasing ECM stiffness and cell proliferation and invasion could be a powerful therapeutic target, especially considering that the increased matrix stiffness can alter the efficiency of chemotherapeutic agents [15]. This interplay between matrix rigidity and cell signaling and growth is dependent on mechanosensing in the cells, a process which requires the force generation power of nonmuscle myosin II as part of the transmission and response to the force signal from focal adhesions and integrins at the cell surface [16–20].

There are three isoforms of nonmuscle myosin II: A, B, and C. Nonmuscle myosin II functions as a hexamer with a pair of heavy chains and two pairs of light chains, regulatory and essential. It is an ATPase capable of converting chemical energy into mechanical work, which is integral to its role in mechanosensing [16, 21]. In addition to its role in mechanotransduction, it has also been shown to be involved in cytokinesis and other cellular processes [22–24]. Force generation is also needed in order for cells to reorganize their surrounding matrix, which contributes to mechanical homeostasis [2, 4, 7]. While we know that myosin II is involved in these processes, limited research has been done looking into the involvement of this motor protein in cancer progression. It has been shown that upregulation or overactivation of myosin IIA is associated with poor prognosis in esophageal [25] and lung cancer [26]. Additionally, in gastric cancer, a decrease in expression of Let-7f, a microRNA that directly binds the 3’UTR of the myosin IIA gene, is associated with an increase in myosin IIA expression and the invasive potential of gastric cancer cells [27]. Finally, tumor tissue in the 3MC induced murine hind leg model of cancer has increased levels of both myosin IIA and IIB compared to tumor associated normal tissue [28]. Given these changes in myosin II regulation in various cancers, and its role in mechanoreciprocity, it could be a strong potential target to break the ECM stiffness/cancer progression feedback loop.

To investigate the role of nonmuscle myosin II isoforms in tumor cell driven remodeling of the ECM, we generated stable myosin IIA and IIB knockdown (KD) MDA-MB-231 cell lines. The morphology was characterized in both two- and three- dimensional culture model systems. We then tested the cell lines for their ability to remodel and constrict a 3D collagen matrix. Complementing the gel compression measurements, we used mechanical testing protocols to measure the stiffness and elasticity of cell populated collagen matrices. This assay allows the direct measurement of biophysical characteristics of the matrix and is similar that used by Wakatsuki, et al [29]. Here we show that loss of myosin IIA blocks the ability of the cells to compress a matrix and results in a matrix with decreased stiffness compared to parental or IIB KD modified matrices. The IIB KD cells are able to compress the collagen gels, but the collagen constructs containing these cells have a different elastic modulus profile than parental cell constructs, indicating that the changes they make to the collagen matrix are not the same as those made by the parental cells. These results indicate that nonmuscle myosin II is involved in matrix remodeling and mechanical homeostasis, making it a potential therapeutic target for blocking the effects of matrix stiffness on tumor proliferation and progression.

## Materials and Methods

### Cell culture

MDA-MB-231 (ATCC, HT-B26) cells were grown and maintained in Minimal Essential Media (MEM) supplemented with 10% FCS, 100 U/ml penicillin, and 100 μg/ml streptomycin (media components were purchased from Sigma Aldrich) in a 37°C humidified 5% CO_2_ tissue culture incubator.

### Generation of Myosin II Knockdown Cell Lines

Lentiviruses were produced in 293T/17 cells as outlined by Tiscornia, et al. [30] using 2^nd^ generation transfer plasmids. Myosin IIA Heavy Chain shRNAs (Cat # RHS4533) and IIB Heavy Chain shRNAs (Cat # RHS4531) were obtained from Openbiosystems (Waltham, MA, USA). After screening all clones, myosin IIA shRNA clone #29467 and myosin IIB shRNA clone #123076 were determined to be isoform specific and produce the most efficient myosin II knockdown. These clones were used in all experiments. For viral infections, MDA-MB-231 cells were seeded at a density of 4X10^5^ cells and allowed to adhere and spread overnight. Two mL of viral stock was added to each culture and virus incubated with cells for 72 hours. Cultures were washed with MEM+10% FCS and allowed to recover for 1 day. For selection and maintenance of MDA-MB-231 cell lines, cultures were fed with MEM+10%FCS containing 5 μg/mL puromycin (Sigma Aldrich). Myosin IIA and IIB KD was verified using Western Blot analysis.

### Immunofluorescence staining

For indirect immunofluorescence staining, MDA-MB-231 control (parental) and MDA-MB-231 myosin II KD cells were fixed and stained for actin and myosin II as described previously [31]. Cells were labeled with affinity-purified rabbit anti-myosin heavy chain IIA or IIB antibody (final dilution 1:1000) as well as TRITC-phalloidin (Sigma-Aldrich, St. Louis, MO, USA Cat # P1951). Alexa 488 goat α-rabbbit secondary antibody (Invitrogen, Grand Island, NY, USA Cat # A11070) was used at a final dilution of 1:1000. MDA-MB-231 cells were mounted in 90% glycerol/10% PBS containing 0.1 M *n*-propyl gallate (Sigma-Aldrich, St. Louis, MO, USA Cat # P3130). Imaging was performed using a Zeiss LSM 510 confocal microscope. A Plan-Apochromat 63x/1.40 Oil DIC M27 objective was used and composite images were constructed from 0.3-μm optical sections. To quantify the fluorescent staining, cells were stained as outlined above, and imaged using a ZEISS Axiovert 40 CFL microscope with a LD A-Plan 20X objective. Quantification was performed using Image J software on at least 25 cells per experiment, across three experiments.

### 3D Morphology

Methods for pouring collagen gels were performed as described in detail previously [31, 32]. Collagen gels were made by suspending 1x10^6^ cells/ml of MDA-MB-231 controls (parental) or MDA-MB-231 myosin IIA or myosin IIB KD in a collagen/MEM solution containing 1.0 mg/ml Type I rat tail collagen. Collagen/cell suspension (1 ml) was poured into Teflon casting molds with a central mandrel and transferred to a 37°C incubator for 1 hour to initiate collagen polymerization. The collagen gel forms (3 mm thick, 3 cm diameter) between the inner wall of the Teflon cylinder and the central mandrel giving rise to a collagen/cell matrix in the shape of a ring (henceforth referred to as a construct). Teflon casting molds were then filled with MEM-10% FCS and incubated for 4 days in a humidified incubator at 37°C with 5% CO_2_. At the appropriate time, collagen gels were removed from the molds, fixed, permeabilized and stained as described for 2D immunofluorescence. Pieces of the collagen gels were cut and processed for staining with TRITC-Phalloidin and affinity purified myosin IIA or IIB antibodies as described above. Alexa 488 goat α-rabbbit secondary antibody was used at a final dilution of 1:1000. Prior to being mounted, gel pieces were soaked overnight in 9:1 glycerol:PBS containing 0.1 M n-propyl gallate. Multi-Photon Laser Scanning Microscopy (MPLSM) was used to image stained constructs for gel compression studies. For 3D cell morphology, a Zeiss LSM 510 was used with an EC Plan-Neofluar 40x/1.30 Oil DIC M27 objective. Composite micrographs were constructed from 0.5-μm optical sections. Analysis of cell morphology was performed using IMARIS image analysis software (Version 8.0 Bitplane, Zurich, CHE). The Filaments function was used to measure the average number of cell protrusions. To calculate the sphericity of the cell bodies, the Surfaces function was used. Surfaces contours were manually drawn and the sphericity calculated by the software. Sphericity compares the surface area of the object, in this case a cell body, to the surface area of a sphere of the same volume. If the object is a perfect sphere, the sphericity would be 1. Elongation factor was calculated using the measurements function in IMARIS. We defined elongation factor as the longest dimension of the object, the cell body, divided by the shortest. The more elongated the cell body, the higher the elongation factor. These morphology characteristics were measured on cells across three separate experiments, with more than 18 cells for each cell type analyzed per experiment. One-way analysis of variance (ANOVA) with a Tukey post-test was performed in GraphPad Prism (GraphPad Software, Inc., La Jolla, CA, USA) to determine the statistical significance of the differences seen.

### Gel Compression

Collagen gel constructs were poured as described above. While in culture, cells organize and compress the collagen, reducing its volume approximately 2 to 5 fold. For gel compression studies (gel thickness), collagen gels were washed with PBS, fixed and removed from Teflon molds after 1 or 4 days of incubation. These time points were chosen to allow the cells sufficient time to compress the matrix. Allowing the gels to incubate for longer periods of time (past one week) does not result in enhanced matrix compression, and the cells within the construct begin to die. The collagen/cell matrix, which is in the shape of a ring, was cut open and three random non-adjacent pieces of the collagen gel cut from each MDA-MB-231 construct, stained with TRITC-Phalloidin and Hoescht 33258 dye (Sigma-Aldrich, St. Louis, MO, USA Cat # 861405), and mounted as outlined above. To determine the thickness of gels cast with only collagen, 40 μl of 1 μm fluorescent beads (Polysciences, Warrington, PA, Cat# 24062) were added to the collagen/MEM solution prior to pouring constructs. Multi-Photon Laser Scanning Microscopy (MPLSM) was used to measure the full thickness of all collagen constructs. For all experimental conditions, constructs were poured in duplicate and a minimum of five measurements were taken from each piece, for a total of 30 measurements per cell type per experiment. The data shown are averaged from three separate experiments. Construct thickness was compared between gels cast from collagen alone, parental and KD MDA-MB-231 cell lines. For experiments using blebbistatin to inhibit myosin II, constructs were fed daily with MEM+10% FCS containing 50 μM blebbistatin ((S)-(-)-blebbistatin, Toronto Research Chemicals) and fixed at 1 and 4 days. Constructs were then processed and measured as described above. Statistical significance was calculated in GraphPad Prism 6 using one-way ANOVA and a Tukey post-test.

### Isometric Tension and Mechanical Measurements

Constructs were poured as described above. After 4 days of incubation, the central mandrel was removed from the Teflon casting mold and the MDA-MB-231 populated construct gently removed from the mandrel before being looped over a triangular hook connected to an isometric force transducer (Harvard Apparatus model 52–9545, South Natick MA) as described previously [31, 32]. The ring is then looped over a horizontal bar which is connected to a stepper motor controlled by a micro-stepping driver as initially described by Kolodney and Wysolmerski [33]. The apparatus used in this study, is very similar to that used by Wakatsuki et al [29], which includes a detailed schematic of the apparatus and construct pouring method. The collagen gels were placed in a 50 ml thermo-regulated organ bath (Harvard Apparatus, Holliston, MA, USA Cat# 760165) containing 20 mM Hepes-buffered MEM/0.4% bovine serum albumin (Sigma Aldrich). Organ baths were maintained at 37°C for the duration of experiments. The triangular hook and stationary horizontal bar over which the construct was looped were set to hold the collagen ring at its original length of 15 mm, which corresponds to half the circumference of the central mandrel. This configuration allows us to apply a defined stretch over a specific time period and to relax the constructs to their original length at the same rate. For mechanical measurements, constructs were hung and allowed to establish a stable basal tension. After establishing a basal tension, constructs were stretched to a 10% strain (1.5 mm) at a rate of 0.5 mm/min, and immediately relaxed to their original length at the same rate. After this initial stretch, constructs were allowed to recover for at least 60 min, or until a stable basal tension developed. Once a stable tension was reached, 2 μM cytochalasin D was added to the organ bath to depolymerize cell actin filaments and abolish basal tension. After basal tension was eliminated (~ 45 minutes) constructs were then subjected to another 10% strain (1.5 mm) at a rate of 0.5 mm/min, and immediately relaxed to their original length at the same rate. Constructs were allowed to recover for 1 hour before being removed from the apparatus. Recovery was included in the mechanical testing for thoroughness as failure in long-term elastic recovery could indicate changes in the properties of the samples not measured by the elastic modulus calculations; alterations in long-term recovery of the samples tested here were not seen. After being removed from the apparatus, samples were snap frozen for determining DNA and myosin II isoform content. This protocol allowed us to determine how MDA-MB-231 cells actively changed the mechanical properties of the collagen matrix after 4 days in culture. It also allows for determination of how elimination of the active actin contractile cytoskeletal contribution alters construct stiffness. To determine the stiffness of the cell altered collagen matrix alone, MDA-MB-231 collagen gels were treated with deoxycholate and subjected to the stretching protocol. This treatment also allows us to determine if other cell components besides the actin contractile cytoskeleton, such as microtubule networks or cell-matrix attachments, contribute to the overall construct stiffness. For deoxycholate experiments, a separate set of constructs, cast on the same day and with the same collagen solution as the initial and cytochalasin D treated constructs, were hung on the apparatus and subjected to the initial stretching protocol outlined above. After 45 min recovery, deoxycholate was added to the organ bath at a final concentration of 0.5% and constructs incubated in the presence of the detergent for an additional 60 min before being subjected to another 10% strain. For all experiments, constructs were hung in duplicate for each treatment type. During each stretching protocol, isometric tension generated by constructs was recorded every second at 5Hz for the duration of an experiment. At the end of each experiment, constructs were removed from the apparatus and snap frozen for DNA and myosin II isoform content analysis. In optimizing stretch parameters, total strains of 5, 10, 15, and 20%, and strain rates of 0.2, 0.5, 0.7, and 0.9 mm/min were tested.

### Estimation of cross-sectional area

Collagen constructs were laid flat on a glass plate and collagen construct width was calculated by taking high resolution digital photographs and measuring the number of pixels across the width of the construct. The thickness of the specimens was determined by Multi-Photon Laser Scanning Microscopy as outlined above (gel compression).

### Measurement of Cell Concentration and DNA Analysis

The final cell concentration within collagen constructs was calculated from standard curves generated using MDA-MB-231 samples of known cell number in increments from 100,000 to 5 million cells. Frozen construct samples were resuspended in 750 μL of 0.1% SDS in PBS and sonicated until homogenous. Samples were diluted using 0.1% SDS in PBS at 1:50 and 1:100 dilutions and 100 μL of each dilution loaded into a microwell plate (Nunc Part No. 237017). Hoescht 33258 stain was added to each well at a concentration of 0.09 μg/mL per well and the plate was analyzed using the Hoescht 33528 protocol on a Modulus Microplate plate reader (Turner Biosystems, Model number 9300–002).

### Calculation of Elastic Modulus

Tension readings were converted from dynes to millinewtons (mN) and plotted against percent strain using Sigmaplot (Version 8.02, Systat Software, San Jose, California, USA). Hysteresis curves were generated by plotting the force readings during stretching and unloading against strain as a function of time. Because each strain is reached twice, once during stretching and once during unloading, the resulting graph starts from zero, reaches 10% strain, and then returns to zero. The upward sweep of each curve is the tension produced during stretching, and the downward sweep is tension during unloading of the sample. To measure the elastic modulus of the constructs, the cross-sectional area must be determined. The cross-sectional area is defined as the area of the sample where the force is applied; in this case, the thickness and the width of the construct (calculated as outlined above). Tension readings, in N, were divided by the cross-sectional area, in m^2^, to determine the stress on the sample in Pascals (Pa; 1Pa = 1N/m^2^). When the stress in Pa is plotted against strain, the slope of the line during the stretch portion of the curve is the elastic modulus of the sample. This slope was calculated using the Linear Regression tool in SigmaPlot, with a confidence interval of 95%. Using the Rule of Mixtures (below), we calculated the contribution of individual components of a sample to the total elastic modulus. For the collagen constructs, there are two main components: the matrix itself and the cells within the matrix.

$$E_{construct}=fE_{cells}+(1-f)E_{matrix}$$

Where E_construct_ is the elastic modulus of the entire construct, determined using the initial stretch of the untreated construct; E_matrix_ is the elastic modulus of the matrix component of the constructs, determined using the DOC treated stretch; and *f* is the volume fraction. Volume fraction is defined as the volume of the component of interest, in this case the volume of the cells, divided by the volume of the complete sample. To calculate the cell volume, samples were stained with TRITC-Phalloidin and imaged using MPLSM for the full depth of the sample. Using the Surfaces function of IMARIS software, the volume of the TRITC channel was calculated. The total volume was calculated using the known frame size of the z-stacks taken. The volume fractions were determined for two full z-stacks for each construct and were consistent between constructs for each cell type. After the elastic modulus of the cell component was determined, the average elastic modulus for individual cells was calculated by dividing E_cells_ by the total number of cells in the construct. In addition to the elastic modulus, we calculated the elastic recovery of samples. For the purposes of this study, elastic recovery is defined as the slope of the curve during the initial recovery after stretching (the first 30 time points after unloading begins). These experiments were performed three separate times, and the statistical significance was calculated using one-way ANOVA with a Tukey post-test.

### Collagen Isolation

Rat tails from previously euthanized animals were obtained from animal quarters. Animals were euthanized under an approved West Virginia University Institutional Animal Care and Use Committee protocol and the tails removed by veterinarian staff. Immediately upon receiving the tails, they were sterilized with 70% EtOH. In a sterile environment, the tails were skinned by snipping off the tip, then making an incision with a scalpel the length of the tail before peeling back the skin. Collagen bundles were then severed at each end of the tail with a scalpel, and pulled from the tail using hemostats. Collagen fibrils were placed in sterile 4°C PBS on ice until collagen from 4–5 large tails had been removed (at least 5 g of collagen). Any excess tissue was removed from the collagen fibrils before placing the collagen in 70% EtOH for one hour on ice. Fibrils were then washed twice more with 70% EtOH before being washed once each with PBS and sterile water. After the collagen was washed, the fibrils were placed in 300 mL of sterile 20 mM acetic acid. The collagen solution was then stirred at a slow rate for 48 hours at 4°C, occasionally mixing with a sterile pipet to ensure complete mixing. After the extraction period, the collagen was centrifuged at 16,000xg for 90 minutes at 4°C. Collagen concentration was determined using SDS-PAGE and stored at 4°C, protected from light.

## Results

### Knockdown of Myosin II Isoforms in MDA-MB-231 Cells

**Fig 1:**
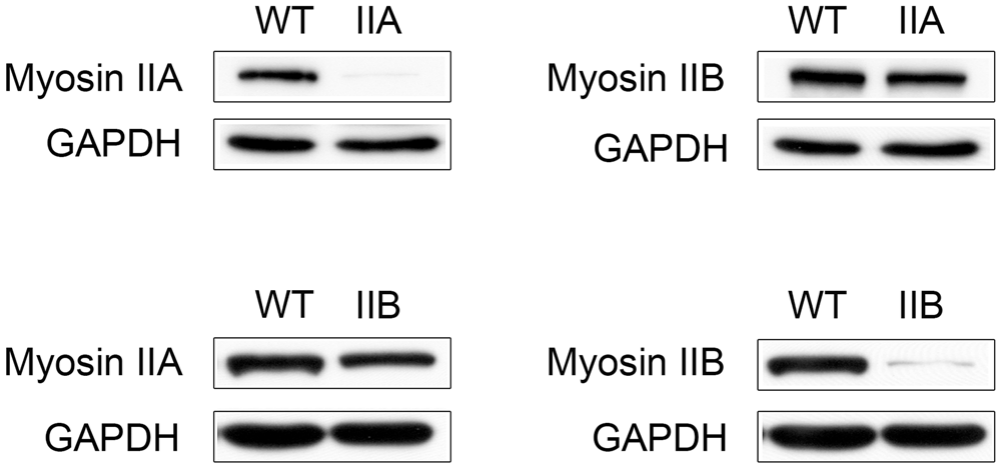
Knockdown of myosin II isoforms MDA-MB-231 cells expressing shRNA targeting either the IIA or IIB isoform of nonmuscle myosin II were analyzed for myosin expression levels; GAPDH was used as a loading control. Greater than 85% knockdown of myosin protein content was achieved in stable cell populations. Myosin isoform levels were assessed for every experiment to verify the level of myosin IIA and IIB knockdown.

It has been established in the literature that MDA-MB-231 cells express the IIA and IIB isoforms of nonmuscle myosin II, but express low levels of IIC not detectable by Western blot [34–36]. In order to test the contribution of each of the major myosin II isoforms in MDA-MD-231 cells, we performed knockdown (KD) of myosin IIA and IIB and assessed what effect myosin KD had on cell morphology (2D and 3D), actin organization, and the ability of MDA-MB-231 cells to compress and organize 3D collagen matrices. Lentiviral shRNA constructs against the two isoforms were screened for specificity and efficient myosin II KD. As shown in Fig 1, each shRNA construct achieved greater than 85% KD of their respective isoforms, while only minimally affecting the non-targeted isoform. Efficient knockdown of myosin isoforms was verified using immunofluorescence (S1 Fig).

**Fig 2:**
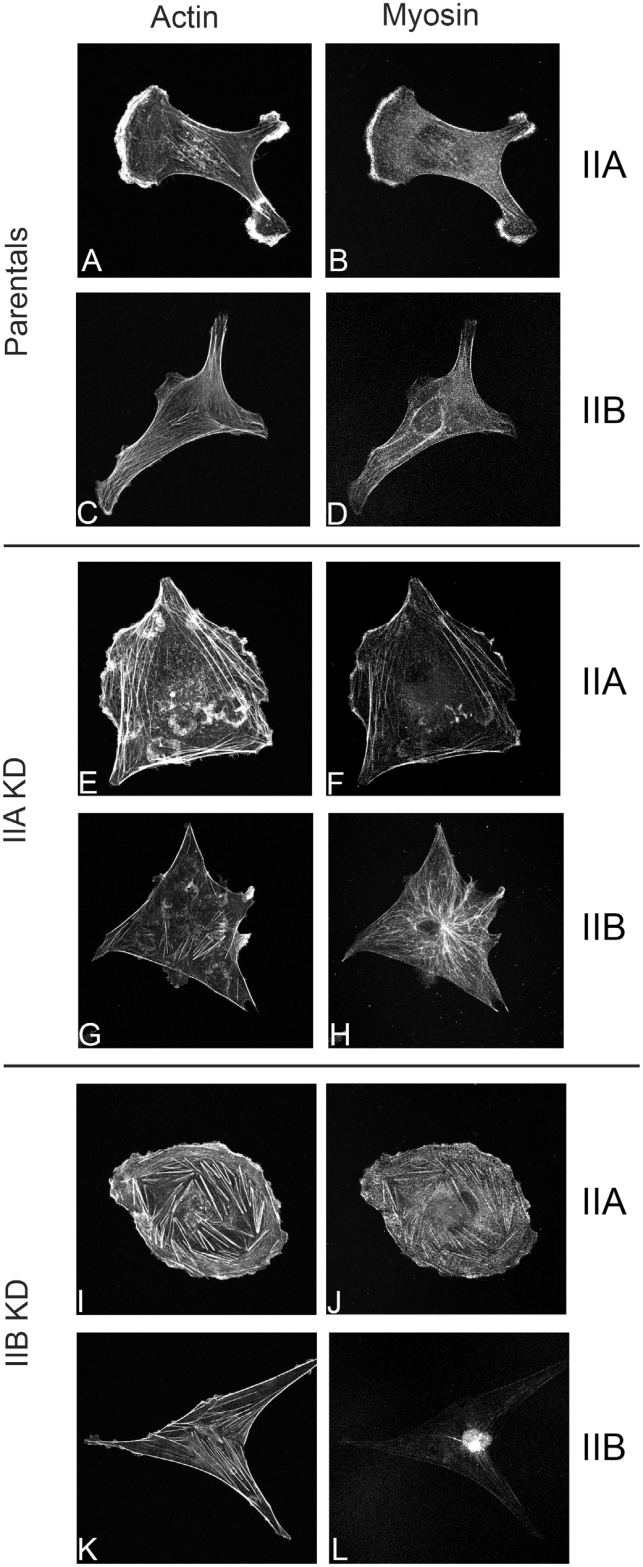
Knockdown of myosin II isoforms induces cytoskeletal changes in MDA-MB-231 cells in 2D Parental (A-D), IIA KD (E-H) and IIB KD (I-L) cells were fixed, permeabilized, and immunostained with affinity purified polyclonal myosin IIA and IIB primary antibodies and TRITC-Phalloidin to visualize actin filaments and myosin localization. In parental MDA-MB-231 cells, myosin IIA **(B)** localizes to stress fibers and the leading edge of cells, while myosin IIB **(D)** had cytosolic, stress fiber, and perinuclear localization. Myosin IIA KD cells had altered actin cytoskeletal structure and were slightly larger than parental controls, while the residual IIA **(E)** in these cells localized to stress fibers and myosin IIB **(H)** localization was slightly affected, displaying a microtubule-like staining pattern, thought there was still an amount remaining largely diffuse throughout the cytosol with some stress fiber and perinuclear localization. Myosin IIB KD cells exhibited a more irregular shape with short, prominent stress fibers, and the residual IIB in these cells exhibited a perinuclear localization **(L)** IIA localization was primarily to stress fibers **(J)**, as in the parental cells.

We next sought to determine if loss of either myosin IIA or IIB affects general cell morphology as well as actin and myosin II distribution. Fig 2 illustrates representative immunofluorescent 2D images of parental controls and myosin IIA and IIB KD MDA-MB-231 cells. F-actin in parental MDA-MB-231 cells was localized to lamellapodia and central stress fibers (Fig 2A and 2C). Myosin IIA co-localized to the underlying actin filaments in lamellapodia and stress fibers (Fig 2B) while myosin IIB was localized to stress fibers and concentrated in the perinuclear region (Fig 2D). Knockdown of myosin IIA caused a disruption in the actin stress fibers of cells, exhibiting prominent stress fibers at the periphery of the cell, and fewer centrally located fibers (Fig 2E). Residual myosin IIA (Fig 2F) was detected at low levels in KD cells where it localized to the actin stress fibers. Knockdown of myosin IIA appears to alter the distribution of myosin IIB, which seems to assume a microtubule like distribution pattern (Fig 2G and 2H). We speculate this might result from IIB binding to a microtubule associated protein. Loss of myosin IIB (Fig 2I–2L) resulted in formation of shorter, thicker stress fibers heavily decorated with myosin IIA (Fig 2I and 2J). These fibers were randomly distributed throughout the cytoplasm. In contrast, KD of myosin IIB for the most part abolished myosin IIB staining associated with underlying stress fibers (Fig 2L). Any remaining myosin IIB exhibited a perinuclear localization (Fig 2L). In 2D, MDA-MB-231 cells showed a variety of morphologies and shapes.

In attempt to generate a microenvironment similar to what cells encounter *in vivo*, MDA-MB-231 cells were cast within 3-D collagen matrices. MDA-MB-231 parental, IIA, and IIB KD cell lines suspended in type 1 rat tail collagen were poured into Teflon casting molds and incubated for 4 days. Over this time period cells spread, organize and compress the collagen matrix. Since neither the cells nor collagen are able to adhere to Teflon, the influence of the casting mold on matrix organization is minimized. Therefore, MDA-MB-231 cell-matrix and cell-cell interactions are primarily responsible for generating the 3-D organized matrix within the Teflon casting mold. After 4 days, collagen constructs were fixed and stained for actin and myosin IIA or myosin IIB.

**Fig 3:**
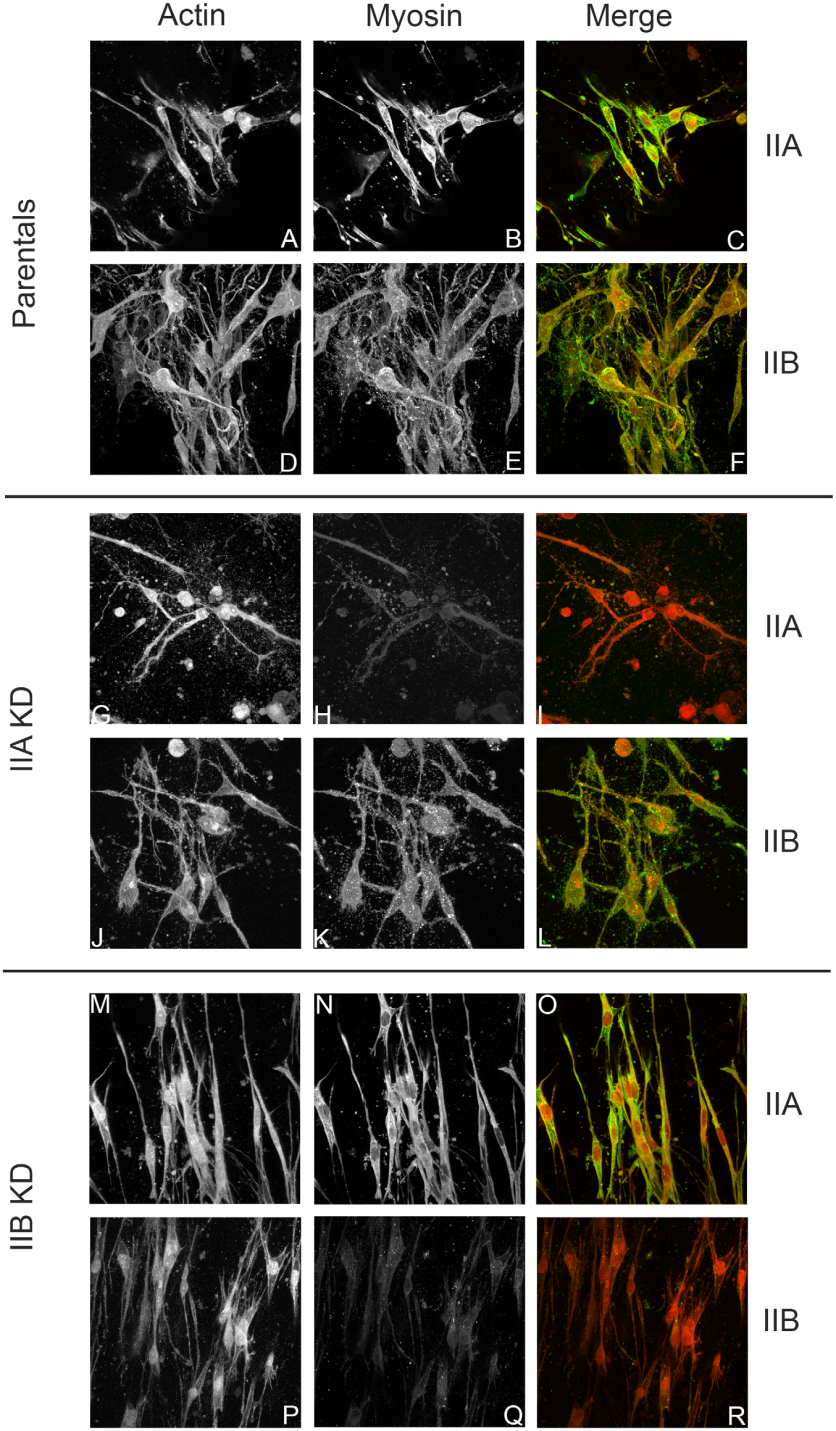
Loss of myosin II isoforms induces morphological changes in 3D Cells were added to a collagen solution, poured into Teflon molds and allowed to incubate for 4 days. Constructs were washed, fixed, permeabilized, and stained with Phalloidin-TRITC and affinity purified myosin II antibodies. Cells were examined using Two Photon Microscopy. **(A-F)** Parental MDA-MB-231 cells in three dimensions had pyramidal cell bodies with multiple projections and significant staining of both IIA **(B)** and IIB **(E)** myosin isoforms, mainly diffuse throughout the cytosol. **(G-L)** IIA KD cells had rounded cell bodies with highly branched and elongated projections in all directions and very little residual myosin IIA **(H)** staining, mostly localized to the cell bodies. The IIB **(K)** in these cells remained diffuse throughout the cytosol. **(M-R)** IIB KD cells were elongated with fewer projections and tended to be localized to a single focal plane, with residual IIB **(O)** localized at cell edges and near the nucleus. For all cell types, myosin isoform localization in 3D was mainly cytosolic.

**Fig 4:**
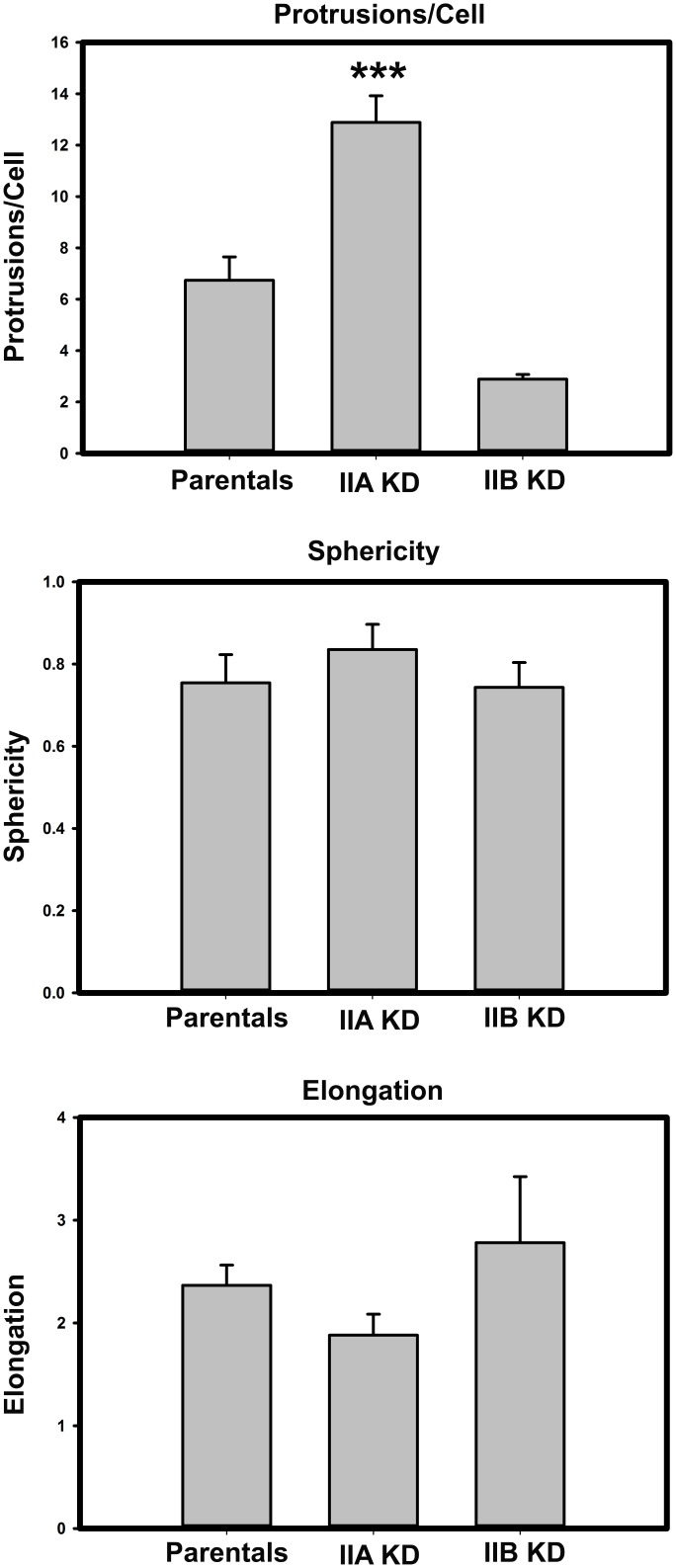
Morphological characteristics of cells lacking myosin II isoforms Collagen constructs containing cells were prepared and imaged as outlined. IMARIS image analysis software was used to quantify the observations made on cell morphology in 3D collagen gels across three separate experiments. **(A)** The average number of protrusions per cell were measured using the Filaments function in IMARIS. Statistical significance was calculated using one-way ANOVA with a Tukey post-test. The difference between parental and IIA KD cell types was significant (p < 0.01), as was the difference between IIA and IIB KD cells (p < 0.001). **(B)** The sphericity of the cell bodies was calculated using the Surfaces function in IMARIS. IIA KD cells were slightly more spherical than parental or IIB KD cells, though the difference was not statistically significant. **(C)** The elongation factor of the cell bodies was calculated using the measurements function in IMARIS. We defined the elongation factor as the measurement of the longest dimension of the cell body, divided by the measurement of the shortest dimension. IIB KD cells had a slightly higher elongation factor than the parental or IIA KD cells, though the difference was not statistically significant.

Fig 3 shows representative images illustrating the morphology and the actin/myosin II distribution of MDA-MB-231 cells in 3D matrices. Parental (Fig 3A–3F), myosin IIA KD (Fig 3G–3L) and myosin IIB KD (Fig 3M–3R) cells exhibit distinct morphology in 3D. Parental cells (Fig 3A–3F) exhibited a rounded or pyramidal like cell body with multiple cell processes extending in various directions and focal planes. Actin (Fig 3A and 3D) was localized to the cytoplasm and cell processes. Both myosin IIA and IIB (Fig 3B 3C 3E and 3F, respectively) co-localize to the underlying actin filaments in the cell body and processes, and also exhibit diffuse staining throughout the cytoplasm. Myosin IIA KD cells (Fig 3G–3L) have a more rounded cell body with numerous slender cell processes extending into several focal planes of the 3D construct. Lack of myosin IIA staining (Fig 3H and 3I), in conjunction with western blot analysis (Fig 1), confirms efficient myosin IIA KD and shows that loss of IIA had little effect on myosin IIB localization (Fig 3K and 3H). In contrast, IIB KD cells within the collagen matrix are long slender cells that lack cell processes, confining IIB KD cells to a single focal plane. Lack of myosin IIB staining (Fig 3Q), in conjunction with western blot analysis (Fig 1B), showed IIB KD had little effect on IIA distribution. The changes in cell morphology in 3D were quantified using IMARIS software (Fig 4). Fig 4A shows the average number of protrusions per cell. Parental cells had 6.7 protrusions per cell, while IIA KD cells had 12.9, and IIB KD cells had 2.9 protrusions. IIA KD cells had significantly more protrusions per cell than either the parental or IIB KD cells. We also calculated the sphericity (Fig 4B) and elongation (Fig 4C) of the cell bodies. IIA KD cells had slightly more spherical cell bodies, and IIB KD cells had slight more elongated cell bodies, though these differences were not statistically significant. These results suggest that myosin II isoforms may regulate how cells are able to interact and organize their surrounding matrices.

### Myosin II Isoforms and 3-D Collagen Gel Compression

Myosin II has been proposed to be critical for mechanotransduction [21], and for generation of cellular forces essential for matrix remodeling. To measure the ability of MDA-MB-231 cells to organize and compress 3-D collagen gels, we developed a gel compression assay. MDA-MB-231 cells were mixed in type I collagen and cast into a Teflon casting mold and allowed to organize and compress the collagen gel for 1 to 4 days, before being fixed and removed from the molds. At this point, the cells have compressed the collagen matrix into a tight ring around the central mandrel of the casting mold. The ring is cut open and the thickness of the collagen constructs measured using microscopy. For determining the thickness of gels cast with only collagen, 1 μm fluorescent beads were added to the collagen/MEM solution prior to casting constructs. Construct thickness was compared between gels cast from collagen alone, parental, and myosin KD MDA-MB-231 cell lines.

**Fig 5:**
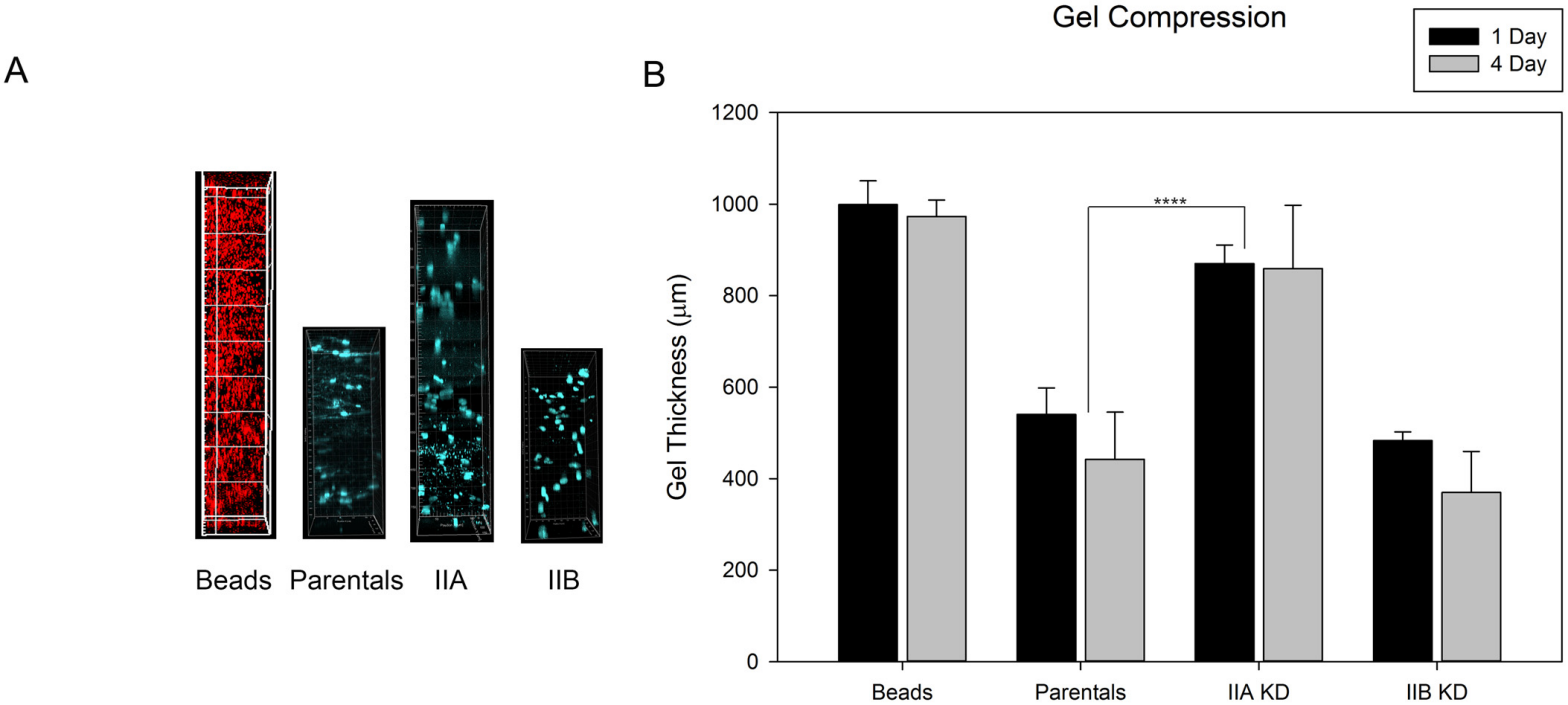
Loss of myosin II isoforms inhibits the ability of MDA-MB-231 cells to compress a collagen gel MDA-MB-231 cells or 1 μm fluorescent beads were mixed with a collagen solution and poured into a Teflon mold with a central mandrel. Constructs were incubated for 1 to 4 days to allow the cells to compress the collagen gel. MDA-MB-231 constructs were washed, fixed, permeabilized, and stained with TRITC-Phalloidin and Hoescht 33258 dye. Cells were examined using Two Photon Microscopy. **(A)** Z-stacks were taken from the top to the bottom of the constructs and the thickness recorded. Representative z-stacks of each cell type, stained with Hoescht 33258 dye to ensure even distribution of the cells through the depth of the collagen gel, are shown. Images were examined using IMARIS software and maximum intensity projections were generated using the Volume function. **(B)**. Data shown are average collagen gel thickness measurements (error bars are SD) from three separate experiments. Parental MDA-MB-231 cells were able to constrict the collagen gel by over 50%, as were IIB KD cells, while IIA KD cells were only able to constrict the gel by 15%. The statistical significance was calculated using one-way analysis of variance with a Tukey post-test on both the 1 and 4 day measurements. The difference between collagen with fluorescent beads alone and parental and IIB KD cell constructs (p < 0.0001) was significant, as was the difference between the beads alone and IIA KD constructs (p < 0.05). In addition, the difference between parental and IIA KD constructs was significant (p < 0.0001).

Examples of representative z-stacks depicting the thickness of collagen constructs generated from collagen alone, parental and myosin IIA and IIB KD MDA-MB-231 cell lines are shown in Fig 5A. Comparing the measured thickness of these different constructs allowed us to calculate to what extent control and KD cells compress a collagen gel. Collagen constructs cast from collagen alone were approximately 1024.7 μm thick 1 day post casting, and after 4 days were measured to be 1023.7 μm thick (Fig 5A and 5B). Parental cells were able to compress the matrix by 50% (540.6 μm) 1 day post casting, and 57% (442.1 μm) 4 days post casting, compared to constructs containing collagen alone. Although the majority of the matrix organization/compression occurs within 24 hours, the process continued for the 4 day duration of the experiment. Myosin IIA KD cells were unable to effectively constrict the collagen construct (Fig 5B). Myosin IIA KD cell were only able to compress the matrix 15.2% (870.2 μm) and 16.1% (858.9 μm) 1 and 4 days post casting compared to constructs cast from only collagen. Parental cells generated 36% and 40% more matrix compression on day 1 and day 4, respectively, compared to the myosin IIA KD cells. These differences were statistically significant (p < 0.0001). Interestingly, we found that the IIB KD cells behaved similarly to parental cells in their ability to organize and compress the collagen constructs. Myosin IIB KD cells compressed the matrix 53% (483.0 μm) and 64% (369.9 μm) after 1 and 4 days, respectively compared to constructs cast from only collagen. This extent of compression was similar to the parental control 50% and 57% 1 and 4 days post casting (Fig 5B). We speculate that the loss of matrix remodeling exhibited by the myosin IIA KD cells could be due to the loss of the force generating capacity needed to physically modify the matrix or the loss of myosin IIA involvement in integrin signaling [37, 38]. Since loss of myosin IIB did not cause significant disruption in matrix compression, our data suggests that myosin II isoforms do not have redundant roles in this cellular process, but rather have separate functions.

In an attempt to inhibit total myosin II function we also measured the ability of parental cells treated with the small molecule myosin II inhibitor blebbistatin to compress a collagen construct. Parental cells treated with blebbistatin were only able to compress the gel 40% (583 μm) after 4 days compared to constructs cast from only collagen (data not shown). Blebbistatin treated constructs have a decreased ability to alter the matrix, however, it is not completely ablated. This suggests that cells may be using a myosin II independent mechanism or we were unable to completely inhibit myosin due to blebbistatin absorbing to the collagen matrix or its degradation in aqueous media.

### Myosin II Isoform Involvement in Isometric Tension of Collagen Constructs

While the gel compression assays are a measure of the amount of matrix remodeling cells are capable of, it does not directly measure changes in the matrix itself. Because matrix stiffness can also independently affect tumor progression, we sought to develop a way to directly measure matrix rigidity and elasticity. To begin to measure this, we first needed a method to measure changes in tension and force production in the collagen constructs as a whole. Using our isometric tension recordings apparatus we were able to measure the tension produced by collagen constructs both at rest and in response to physical strain. Collagen constructs were poured into the Teflon casting molds, removed after 4 days and hung from isometric force transducers, as described in Wakatsuki, et al [29]. After establishing a basal tension, constructs were stretched to 10% strain (1.5 mm) at a rate of 0.5 mm/min and immediately relaxed to their original length at the same rate. The resulting tension was measured and plotted against strain in hysteresis curves. In these graphs, the upward sweep of the curve represents the tension produced by the construct during stretching, while the downward portion is the recovery of the sample during unloading. These hysteresis curves are used to gain insight into the stiffness and elasticity of the constructs.

**Fig 6:**
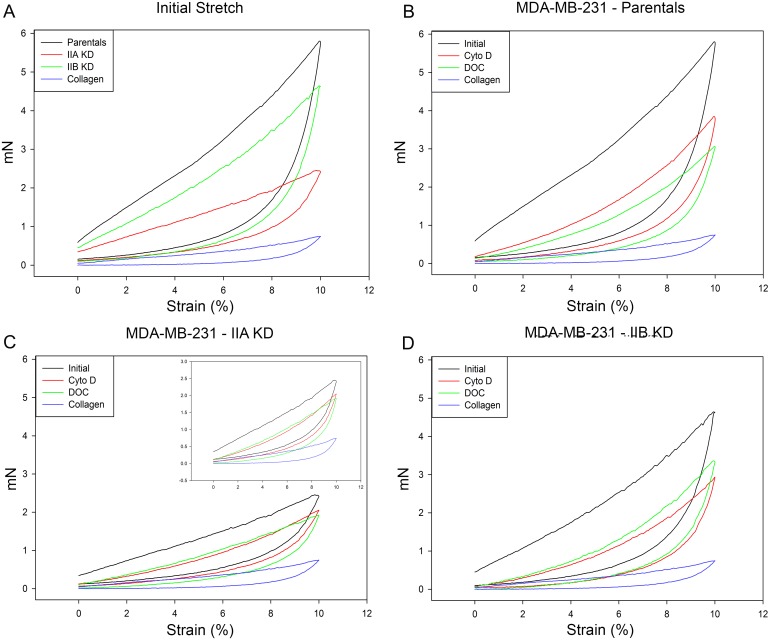
**Isometric tension development of MDA-MB-231 cells** Representative isometric tension tracings from a single experiment, plotted as mN stress versus percent strain, are shown for each of the following: **(A)** readings generated by parental cells (black), IIA KD cells (red), IIB KD cells (green), and collagen alone (blue) during an initial 10% stretch of untreated constructs. Myosin KD cells exhibited a lower response to strain compared to parental constructs, especially IIA KD cells, while collagen alone constructs had a negligible response to the strain. **(B)** Parental cell constructs with no treatment (black), treated with cytochalasin D to disrupt the active actin component (red), or treated with deoxycholate to remove the cell completely (green). These readings are compared against collagen alone (blue). **(C)** IIA KD cell constructs with no treatment (black), treated with cytochalasin D (red), or treated with deoxycholate (green) are compared to collagen alone (blue). A similar pattern in treatment responses was seen to that of the parentals. **Inset** IIA KD cell construct force measurements are shown with a reduced y-axis scale to highlight the differences between treatment conditions. **(D)** IIB KD cell constructs with no treatment (black), treated with cytochalasin D (red) or treated with deoxycholate (green) are compared to collagen alone (blue) For all cell types **(B-D)** treatment with either cytochalasin D or deoxycholate greatly disrupted the response of the collagen constructs to stretching, with deoxycholate having a greater effect, indicating that cell components other than the actin contractile component contribute to the overall construct response to mechanical strain.

Fig 6A shows the hysteresis curves, plotted as mN versus percent strain, for collagen alone (blue line), parental (black line), myosin IIA KD (red line), and IIB KD (green line) MDA-MB-231 collagen constructs during stretching and unloading from a single, representative experiment. Parental samples (the black line in Fig 6A) show a marked increase in tension during the period of stretching, a 10-fold increase in tension at the maximum stretch. Comparing the parental samples to the collagen alone (the blue line in Fig 6A), it is evident that the cells are significantly altering the collagen matrix. The collagen alone constructs are very loose compared to the much tighter network generated by parental cells. The loose, unorganized matrix produces a decreased tension signature in response to strain. The myosin IIA KD and IIB KD samples (the red and green lines in Fig 6A, respectively), also show a decreased response to physical strain when compared to the parental samples, indicating that they do not respond to physical stress in the same way as the parental cells. Interestingly, the MDA-MB-231 samples do not return to the same baseline tension after unloading, indicating the collagen constructs are not perfectly elastic. The changes in collagen construct tension between the various cell lines correspond well to the differences seen in the gel compression assay (Fig 5). Parental cells are able to compress the collagen and generate a significant tension in response to increased strain, while myosin IIA KD cells are unable to properly modify their surrounding matrix resulting in a 2.4 fold decrease in peak tension generation in response to applied strain. Myosin IIB KD cells exhibit a tension response to strain similar to that seen in parental cells, a 10 fold increase from baseline at the maximum stretch. The distinct tension profiles in response to strain for these cell lines suggest differences in the structural arrangement of the matrix, resulting in lower tension generation for less organized, looser constructs (myosin IIA KD cells). This interpretation is in agreement with data obtained from gel compression assays (Fig 5).

The isometric tension measurements examined thus far measure the collagen construct as a whole, i.e. cells and the microenvironment generated by modifying their surrounding matrix. By eliminating certain components of the constructs, we can determine what role(s) each component plays in matrix organization and tension generation. For example, treating the constructs with cytochalasin D, an actin disrupting agent, before subjecting the construct to mechanical testing allowed us to compare the applied strain response produced in cells with an intact actin cytoskeleton (active contractile cell) with those from which the actin cytoskeletal contributions of the cell have been eliminated. This treatment provided information about the active contractile force generated by the cells within the construct. Furthermore, treating constructs with the non-ionic detergent deoxycholate uncoupled cell membrane matrix interactions (by dissolving the cell) leaving only the collagen construct, devoid of cells, to respond to the applied strain. The cell bodies themselves and their attachments to the matrix may contribute to tension simply because their attachments act as an anchor, prohibiting the collagen fibers from stretching as far as they could without these attachments. The tension produced in response to strain after removing cells with detergent allowed us to measure only the inherent contribution of the matrix to the construct rigidity and elasticity.

Fig 6B shows representative hysteresis curves of untreated parental control constructs, as well as parental constructs treated with cytochalasin D and dexoycholate. Cytochalasin D treated constructs (red line in Fig 6B) have a much lower baseline tension compared to untreated controls, 0.19 versus 0.59 mN. Treatment with deoxycholate reduces the baseline even lower to 0.12 mN. As shown in Fig 6B, parental cells generate a peak tension of 5.8 mN in response to applied strain. Disruption of actin filaments resulted in a 1.5 fold (to 3.8 mN) reduction in tension at max stretch, indicating an intact actin/myosin cytoskeleton was needed to generate tension in response to applied strain. Incubation of constructs in the presence of deoxycholate further reduced the peak tension response by 1.3 fold (to 2.9 mN). Parental control constructs cast with MDA-MB-231 cells containing both myosin IIA and IIB generate a peak strain tension 8 fold higher (5.8 mN) than constructs cast with collagen alone (0.75 mN). Both myosin IIA KD (Fig 6C) and IIB KD (Fig 6D) showed a similar pattern of changes in tension production after treatment with cytochalasin D or deoxycholate as the parental constructs. The myosin IIA KD constructs (Fig 6C) generated a 2.4 fold lower peak tension than control constructs indicating the IIA KD cells were unable to stiffen the matrix in a manner comparable to parental cells. After treatment with cytochalasin D and deoxycholate (Fig 6C, red and green curves, respectively), the hysteresis curves indicate the matrix has been organized by the IIA KD cells to impart a structural rigidity to the construct 2.5 fold stiffer than constructs cast from collagen alone; though the IIA KD generated matrix is still 2.9 fold lower than parental controls. This implies that even though the constructs are looser and larger (width and depth) than controls, the IIA KD cell still have the ability to modestly compress and organize the matrix. Myosin IIB KD (Fig 6D) constructs more closely approximate parental control responses, however, the hysteresis curves show that myosin IIA alone (the remaining isoform in IIB KD cells) is not capable of generating the same matrix stiffness as exhibited by parental controls (Fig 6B).

These results, in conjunction with the gel compression studies, indicate that both myosin IIA and IIB are needed for MDA-MB-231 cells to respond to applied stress and organize 3D matrices. Our data points to myosin IIA as the prominent myosin II isoform regulating matrix organization/compression, since loss of IIB has only minimal effects on 3D matrix compression and stiffness. However, myosin IIA is unable to completely compensate for the loss of myosin IIB, indicating that both isoforms are necessary for these cell processes.

### Myosin II Involvement in Matrix Rigidity and Elasticity

**Fig 7:**
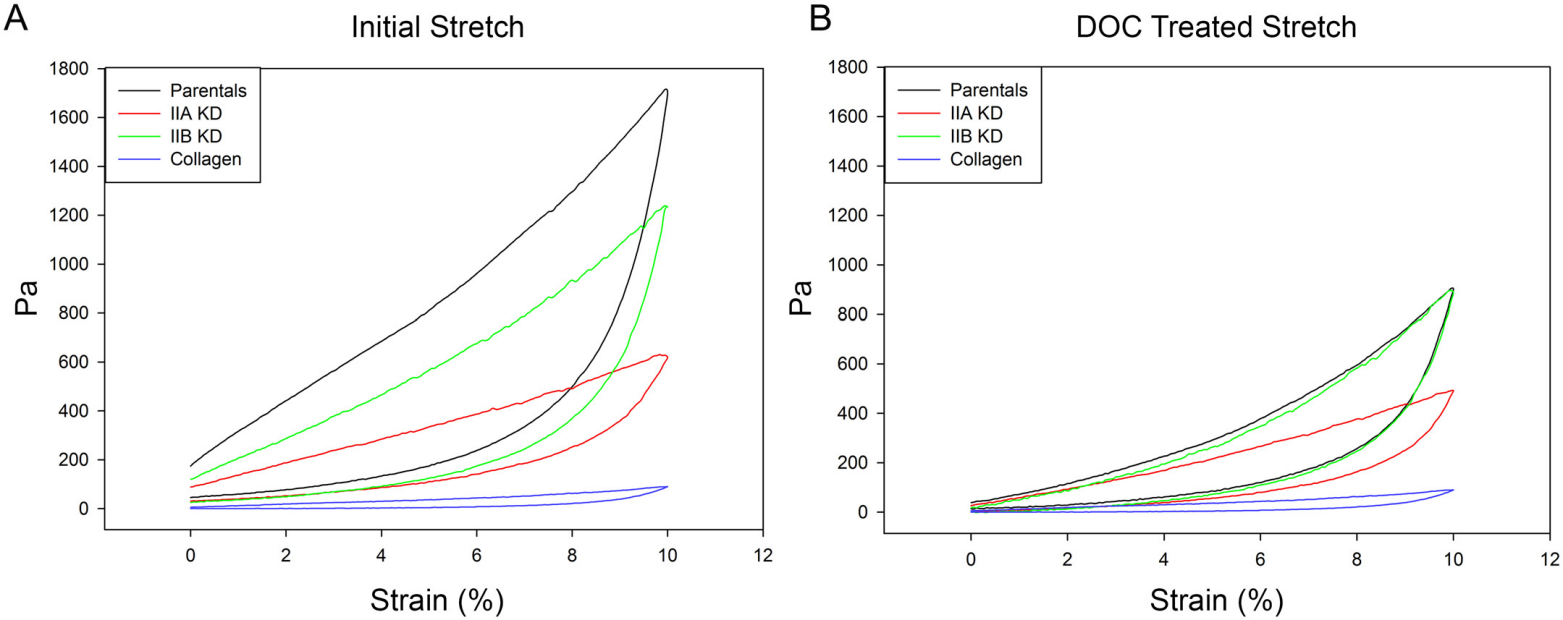
Matrix rigidity of MDA-MB-231 cells Representative collagen construct stress calculations from a single experiment for the initial, untreated stretch (A) and the deoxycholate treated stretch (B) plotted as Pa versus percent strain. For both, myosin II KD cell constructs had a decreased stiffness in response to strain. Because the stiffness calculation takes the cross sectional area of the constructs into account, and the IIA constructs are much larger, the difference between the parental and IIA KD constructs is enhanced when compared to the differences seen when the strain response is plotted as mN versus Strain (Fig 6). Loss of myosin IIA had a more significant effect on force in response to strain than did loss of myosin IIB.

In order to calculate the elasticity of a sample, the stress generated by the construct, rather than the recorded tension, must be determined. Stress is defined as force divided by the cross-sectional area of the sample and is reported as Pascals (1 Pa is equal to 1 N/m^2^). The cross-sectional area of the samples in this study was calculated using the width of the collagen construct multiplied by the thickness of the construct, which corresponds to the area of the construct the strain is acting upon. The cross-sectional areas for the various cell lines differ due to differences in their ability to alter and compress the matrix. Fig 7A shows representative hysteresis curves corrected for the differences in cross-sectional area for parental, myosin IIA and IIB KD MDA-MB-231 cells. The differences between cell lines are highlighted when the cross-sectional area is taken into account. This is especially evident in the IIA KD constructs (the red line in Fig 7A), which exhibits a 64% reduction in peak tension in response to mechanical testing compared to parental constructs and a 50% reduction when compared to myosin IIB KD constructs. These results show myosin II is an essential player in development of matrix stiffness and suggest myosin IIA is more involved in matrix organization than IIB.

Once the stress (force/cross-sectional area) has been calculated it is used to determine the elastic modulus of the construct. Constructs are subjected to mechanical testing and hysteresis curves plotted as Pa versus strain. The slopes of these hysteresis curves are used to calculate the elastic modulus of the sample. This calculation comes from the following equation:

σ=Eε

Where ε is the strain on the sample, σ is the stress of the sample, and E is the elastic modulus of the sample. Because the elastic modulus is defined as stress (Pa) divided by strain (unitless) it has units in Pa. The stress-strain curves for representative experiments of all cell types, as well as collagen alone, are presented in Fig 7A. Similar to the results obtained from the tension versus strain curves (Fig 6), we found major differences in the mechanical properties between constructs cast with collagen alone and constructs cast with MDA-MB-231 cells. Table 1 shows the calculated Construct elastic modulus (organized/compressed collagen matrix containing cells) and Matrix elastic modulus (samples treated with detergent to remove the cells) for parental, myosin IIA and myosin IIB KD MDA-MB-231 cells. Parental constructs had an elastic modulus of 9.22 Pa, 23-fold greater than for constructs cast from collagen alone (0.40 Pa). Myosin IIA KD cell constructs had an elastic modulus of 3.42 Pa, a 2.7 fold decrease from parental controls and 8 fold increase over collagen alone. Myosin IIB KD constructs had an elastic modulus of 7.20 Pa, more closely approximating parental control constructs. These numbers are in agreement with the differences in the ability of cells to alter the collagen gel as measured by gel compression (Fig 5B).

**Table 1:** Elastic modulus of collagen constructs seeded with MDA-MB-231 cells.

Cell Type	Construct Modulus (Pa)	P Value	Matrix Modulus (Pa)	P Value
Parentals	9.22 ± 0.61	NA	4.38 ± 0.50	NA
IIA KD	3.42 ± 0.34	<0.001	2.39 ± 0.37	<0.05
IIB KD	7.20 ± 0.78	ns	4.73 ± 0.53	ns
Collagen	0.40 ± 0.03	<0.0001	0.40 ± 0.03	<0.001

Shown are the averaged elastic moduli (± SEM) for constructs across three separate experiments

It is also important to understand construct mechanics during recovery (unloading) from the strain. For the purposes of this study, we defined elastic recovery as the slope of the hysteresis curve during the initial 30 seconds of sample unloading. As shown in Table 2, the differences between cell types are evident and in agreement with our gel compression data and the elastic modulus: parental cell constructs had an elastic recovery of 38.61 Pa, IIA KD constructs 15.89 Pa, and IIB KD cells 31.86 Pa (Table 2). The difference between collagen alone and collagen containing cells is evident here as well; IIA KD constructs, which are unable to efficiently alter the matrix, produce a 8.3 fold greater elasticity than collagen constructs alone, which exhibited an elastic recovery of 1.92 Pa.

**Table 2:** Elastic recovery of collagen constructs seeded with MDA-MB-231 cells.

Cell Type	Construct (Pa)	P Value	Matrix (Pa)	P Value
Parentals	38.61 ± 1.05	NA	19.65 ± 1.32	NA
IIA KD	15.89 ± 2.19	<0.001	10.70 ± 1.56	<0.01
IIB KD	31.86 ± 4.40	ns	18.77 ± 1.55	ns
Collagen	1.92 ± 0.11	0.0001	1.92 ± 0.11	<0.0001

The elastic recovery, here defined as the slope of the initial recovery of the construct after stretching, was calculated for each cell type. Shown are the averaged (±SEM) elastic recovery for constructs across three experiments.

### Myosin II Isoforms Play Different Roles in Matrix Arrangement and Cellular Response to Strain

The elastic moduli calculated above represent the collagen construct as a whole (Construct Modulus), both matrix and cell component, and that of the cell modified matrix alone after cells are removed using detergent (Matrix Modulus). While cells may be arranging the matrix in a similar way, the cells’ response to physical stress may be different, especially if myosin II isoforms play different roles in matrix interactions and cellular force generation. Using the Rule of Mixtures it is possible to separate the contribution of the matrix and cells to overall construct elasticity. The Rule of Mixtures states that:

$$E_{construct}=fE_{cells}+(1-f)E_{matrix}$$

In the above equation *E*_*construct*_ is the elastic modulus of the untreated samples, *E*_*matrix*_ is the elastic modulus of the constructs treated with deoxycholate (Fig 7B) and *f* is the volume fraction. The volume fraction is defined as the volume of the component of interest (cells), divided by total volume of the construct. For this study, the volume of the cell component of the constructs was calculated using microscopy and divided by the total volume of the image frame. Myosin IIA KD cells had a smaller volume fraction than IIB KD or parental cells (0.014 for the IIA KD cells versus 0.017 and 0.02 for the parental and IIB KD cells, respectively) because the total volume of the construct is greater due to the fact that the IIA KD cells are unable to constrict the matrix (870 μm for the IIA KD cells versus 540 μm for the parental cells). However, the overall cell volume between the cell types is not significantly different (about 9 x 10^6^ μm^3^). For calculations such as this, it is important that the cells be evenly distributed throughout the matrix. As can be seen in Fig 5A, these cells are well distributed and not clustered in one region of the construct. For parental constructs, the cell modulus was determined to be 286.26 Pa. Myosin IIA KD cells had a modulus of 52.89 PA, and IIB KD cells had a modulus of 107.43 Pa (Table 3). The parental cell elastic modulus was 5.4 fold greater than myosin IIA KD total cell elastic modulus and 2.7 fold greater than myosin IIB KD cells. This is consistent with the changes in matrix remodeling ability of the different cell lines, again suggesting that myosin IIA assumes a greater role in organizing/compressing the microenvironment than myosin IIB.

**Table 3:** Calculated elastic modulus of MDA-MB-231 cells within collagen constructs.

Cell Type	Total Cell Modulus (Pa)	P Value	Single Cell Modulus (μPa)	P Value
Parentals	286.26 ± 30.99	NA	189.25 ± 20.68	NA
IIA KD	52.89 ± 17.28	<0.01	36.78 ± 13.38	<0.01
IIB KD	107.43 ± 23.47	<0.01	79.84 ± 24.48	<0.05

The Construct and Matrix moduli of constructs from each experiment were used to calculate the Total Cell Modulus. This was then divided by the cell number, determined by a DNA assay for each experiment, to calculate the Single Cell Modulus. Shown are the calculated total and single cell moduli, averaged across three experiments (± SEM).

Once the elastic modulus of the cell component of the constructs has been calculated, dividing the total elastic modulus by the total number of cells in the constructs will determine the average elastic modulus of each individual cell (Single Cell Modulus; Table 3). The number of cells in each construct was determined to be between 1.3 and 1.8 million cells for each cell type. The single cell elastic modulus was calculated as 189.25 μPa for parental cells, 36.78 μPa for IIA KD cells, and 79.843 μPa for the IIB KD cells (Table 3). Interestingly, the total cell elastic modulus calculated for the parental cells and the IIB KD cells (286.26 and 107.435 μPa, respectively, Table 3) show a divergence with what was predicted based on the overall construct elastic modulus (9.22 vs 7.20 Pa respectively; Table 1). This can be explained when comparing the matrix elastic modulus (deoxycholate treated stretches) for the two cell types. While the overall construct modulus is higher for the parentals than IIB KD, the matrix elastic modulus for the parental constructs is actually lower than that for the myosin IIB KD (4.38 Pa versus 4.73 Pa). This suggests the myosin IIB KD cells arrange and compress the collagen matrix in a different way than parental cells, creating a stiffer construct. It is possible that IIB is restricting or inhibiting myosin IIA’s ability to transmit force across the membrane and maximally organize the matrix. We speculate that upon loss of myosin IIB, myosin IIA is able to transmit force across the membrane more efficiently yielding a stiffer, more rigid matrix.

Our data also clearly show that cells containing only myosin IIA (IIB KD cells) produce 2.2 fold greater tension within the construct than cells expressing only myosin IIB (IIA KD cells). In addition, these data suggest myosin IIA is the major force producing motor protein in the parental cells, since cells lacking IIA had a cell elastic modulus 5.1 fold less than parental cells, while those lacking IIB had an elastic modulus only 2.4 fold less. Nevertheless, both motor proteins are essential for MDA-MB-231 cells to achieve maximum tension production. Thus, calculating the elastic modulus of the total construct, the matrix, and the cells allows one to analyze the mechanical properties of the constructs in a more quantitative way than the gel compression measurements; these calculations are useful in making comparisons to elasticity and rigidity measurements under various experimental conditions or even whole tissues. Taken together, these results suggest that myosin II isoforms have separate roles in the generation and maintenance of cellular stiffness and in their ability to alter and guide matrix organization and stiffness. Myosin IIA is especially important for matrix remodeling and elasticity. These cellular characteristics, due to altered mechanoreciprocity in tumors, may have a drastic impact on tumorigenesis.

## Discussion

Matrix rigidity has been shown to stimulate tumor growth and metastasis [2, 4, 5]. It is known that actomyosin contractility is needed for tension induced cell proliferation in epithelial monolayers [39] as well as ROCK induced tissue changes and cell hyperproliferation in an induced ROCK model of murine cancer [40]. However, the importance of cell motor proteins, such as myosin II, in this process has previously not been directly investigated. Here we show that the myosin II isoforms, IIA and IIB, are involved in cell mediated matrix reorganization, cellular stiffness, and cell mediated changes to matrix stiffness. Loss of myosin IIA especially has a drastic impact on cell mediated matrix reorganization and resulting alterations to matrix stiffness. This could be part of the explanation as to why myosin IIA upregulation has been shown to be correlated with poor prognosis in several types of cancer [25–27] if IIA is needed for matrix alterations that are important for tumor development and later invasion and metastasis [10, 41].

In this study, we measured the matrix remodeling ability of cells with a collagen gel compression assay using Teflon molds. This is similar, on the surface, to the gel contraction assay that has been used for a variety of cell types as a measure of cell mediated matrix reorganization [42]. In fact, Yu et al. [43], found that the phosphorylation state of myosin light chain did not have an effect on cancer cells’ ability to alter the matrix in the gel contraction assay. However, there are several differences between the gel contraction assay as used in past studies, and the gel compression assay used here that make the two difficult to compare. In a typical gel contraction assay, cells are suspended in a 3D matrix, usually collagen, and poured into a multi-well plate. After the collagen has solidified, the gel is detached from the well and changes in the diameter of the resulting matrix disk are measured over time [42]. In such an assay, there is a strong possibility that the plastic of the multi-well plate may impact the behavior of the cells, through interaction with the collagen or the cells themselves. In the development of the assay used here, Teflon was chosen specifically because it is less likely to interfere with the cell mediated alterations to the matrix than a substrate such as plastic, which can interact with collagen. In the gel contraction assay, once the gel is detached from the bottom of the plate it may roll in on itself or undergo other physical contortions that alter the diameter measurements. Also, changes in the thickness of the gel are not measured in the gel contraction assay, generally only changes in the diameter, circumference, or area of the collagen are measured [44]. While the data presented in this paper focus on the thickness, there was a significant difference in total volume of the collagen gels, and this difference is taken into account in the cell elastic modulus calculations. While we have shown these changes, we can only speculate on how changes in myosin status of the cell are translated to the matrix without further study. A likely candidate is the α5β1 integrin, which has been shown to be involved in the development of traction forces and myosin II activation [16, 37, 38, 45]. In our gel compression assay, we show that loss of the IIA isoform of nonmuscle myosin II has more significant effect on the cells’ ability to modify a surrounding matrix compared to loss of the IIB isoform. This effect could be due to the differences in myosin II isoform kinetics [46, 47] or the differences seen in isoform response to mechanical strain [48]. In addition to the known differences in isoform enzyme activity, it has been shown that myosin IIA and IIB have different activation patterns in durotaxis, the phenomenon of cells migrating from a soft matrix to a stiffer one. Raab et al. [49] showed that the IIA isoform is diffuse in mesenchymal stem cells on soft 3D polyacrylamide matrices, but localizes to oriented stress fibers in cells in stiff matrices. This localization is followed by IIB polarization to the rear of the cell. The loss of IIA may prevent the proper localization of the IIB isoform in cells suspended in 3D matrices, blocking the cells’ ability to efficiently use the remaining myosin isoform to interact with the collagen matrix.

There are several methods to measure the elasticity of cells alone or engineered cell/matrix constructs. A popular method in the literature is Atomic Force Microscopy (AFM) [50, 51]. However, AFM was designed for high-resolution imaging of the topography of specimens. For this purpose, the cantilevers used in AFM are extremely compliant and the tip size is typically in the nanometer range [52]. For many studies using AFM to measure stiffness of biological specimens, a glass bead is attached to the end of the tip to prevent the small tip from puncturing cells or slipping into the pores of a 3D matrix, which would confuse the measurements [50, 51]. However, the weight of the glass bead alters the spring constant of the cantilever, and this change must be precisely corrected for in calculations. AFM has also been used to measure elasticity of whole tumors [51]. In the case of large specimens such as this, AFM must take many measurements of small sections of the sample and then the average elastic modulus is calculated. Using AFM, it is also difficult to measure changes in sample elasticity over time or in response to various treatments or conditions. In developing the force conditioning model used here, we strove to design the stretching protocol in such a way that we could measure the global response of collagen constructs to stress over time. Putting the entire sample under strain allows us to calculate how the constructs, and the cells within them, dynamically respond to physical stretching. Additionally, the protocol is non-destructive, allowing for multiple stretches of the same sample under different treatment conditions. Combined with the drawbacks to AFM previously discussed, we determined that the stretching assay was the better model for what we wanted to measure. Studies of whole tumors using AFM calculated an elastic modulus in the kPa range [51], while we calculated an elastic modulus in the Pa range. This large difference is possibly due to the more complicated matrix composition in a whole excised tumor, as well as to differences in the method of measurement.[29, 53–57]. It is also important to note that AFM measures the stiffness of a single point of a specimen in a single point in time; these numbers are often averaged, however that still does not allow for a dynamic response from cells. The stretching assays do allow for this dynamic response on a global scale. The elastic moduli calculated using the stretching assays attach a solid number to the matrix alterations that are indirectly measured in the gel compression assay, and both assays agree that myosin IIA is necessary for efficient gel compression and development of a stiff, yet elastic, matrix. The calculated cell modulus for the IIA KD (52.89 Pa for total cell modulus, 36.78 μPa for the single cell modulus) was also significantly lower than that for parental cells (286.26 Pa and 189.25 μPa), indicating that IIA is necessary for cellular stiffness as well as cell generated matrix changes.

The stiffness of individual cells seeded on glass or plastic substrates, as is used in AFM measurements, would likely be significantly higher than cells seeded in collagen, due to the stiffness of the substrate. Also, due to the nature of AFM measurements, the placement of the indentation tip can have a significant impact on the ultimate stiffness measurements since different cell components have different inherent mechanical properties [53]. Additionally, the spring constant of the cantilever and the geometry of the tip are important in using AFM to calculate sample stiffness [54]. This study used a relatively low starting concentration of collagen (1 mg/mL). Adding additional matrix components, such as laminin, or starting with a higher collagen concentration, could significantly increase the stiffness of the matrix, which could ultimately affect the cellular stiffness as well, due to mechanoreciprocity. Other studies using a stretching method to measure stiffness used contractile fibroblasts, which are much stiffer than epithelial cells [29, 55–57]. The assay used here relied on the mammary epithelial cells alone to generate the stiffness of their surrounding matrix. The elastic modulus of a matrix containing stromal cells such as fibroblasts, which are more contractile than epithelial cells, would likely be much stiffer than a matrix containing epithelial cells alone. This speculation is supported by the fact that fibroblasts compress the collagen to a greater degree than the breast epithelial cells (data not shown). The addition of stromal cells, particularly cancer associated fibroblasts, to this assay, while beyond the scope of this work, could provide insight into how various types of cells associated with tumors contribute to the overall tumor stiffness.

The changes in matrix stiffness between parental and myosin II KD cells were as expected from the gel compression assays, however, the cell modulus, both total and individual, for the IIB KD cells was much lower than predicted based on the gel compression or total construct modulus. The elastic modulus of the IIB KD constructs was calculated to be 7.20 Pa, while the parental was 9.22. Based on these numbers, it was predicted that the IIB KD total cell modulus would be much closer to the parental value of 286.262 Pa (189.25 μPa for single cell modulus) than the 107.435 Pa (79.84 μPa single cell modulus) value that was calculated. Part of this unexpected result is explained in the elastic modulus values of the two different matrices (the deoxycholate treated samples). For those stretches, the IIB KD modulus was higher than that of the parental, 4.73 Pa versus 4.38 Pa, respectively. This indicates that in the IIB KD cell constructs, the matrix is responsible for a relatively large proportion of the overall elastic modulus while the cells themselves contribute little. In these cells, myosin IIA alone is sufficient to arrange a collagen matrix, but not for the cells themselves to respond to a dynamic physical strain. This could be due to the different responses of the two isoforms to mechanical loads. Myosin IIB has been shown to have different ADP release kinetics under mechanical loads, and to be more sensitive to such mechanical changes than IIA [48]. Thus, loss of IIB may prevent the cells from being able to respond to mechanical stress because the remaining IIA isoforms does not respond to mechanical loading in the same way. Here we used the rule of mixtures to calculate the contributions of the different construct components, cells and matrix, to the overall elastic modulus. This method has been used in other studies and is known to have limitations [55–57]. The calculated cell modulus using the rule of mixtures from an unidirectional strain, as used here, is somewhat cell distribution and orientation dependent [55].[55] Since the different cell types used in this study have similar, random, orientations, this effect would not have a significant bearing on the differences seen here, though it could affect the absolute calculated elastic moduli. In addition, there is a need to compensate for the voids left when the cells are removed using deoxycholate [56]. However, the effects are minimal at lower cell concentrations, below 10x10^6^ cells/mL, where the voids do not disrupt the continuity of the matrix. The number of cells in the constructs used here is well below that threshold.

In general, the results shown here indicate that myosin IIA is critical for matrix rearrangement while loss of IIB had a less intense effect on the ability of cells to organize a matrix, though the cell elasticity is affected. These differences in isoform behavior could be due to differences in their kinetics. Myosin IIB has a much higher affinity for ADP, and a slower release rate, which means it spends more of its time bound to actin than does IIA [46, 47, 58]. The two isoforms also have different means of regulation and interactions with other cytosolic proteins that alter myosin actin binding and ATPase activity that could explain the differences seen here [59–61]. Additionally, the two isoforms respond differently under mechanical loads, with myosin IIB showing enhanced mechanosensitivity [48]. Finally, it has been shown in mesenchymal stem cells that myosin IIA localizes to stress fibers of cells in stiff 3D matrices prior to IIB polarization to the rear of the cell, both of which precede migration [49]. If these observations hold true in MDA-MB-231 cells, it could provide a partial explanation for the results seen here. Cells lacking myosin IIA are unable to significantly impact their surrounding matrix. If IIA localization to stress fibers is needed for proper IIB polarization, then IIB alone is unable to generate the force needed on the actin cytoskeleton to arrange the collagen. On the other hand, cells lacking the IIB isoform are able to organize the collagen matrix. In these cells, the IIA isoform localizing to the stress fibers may be enough for the cells to interact with and rearrange the collagen matrix, but not for the cells themselves to generate tension in response to strain. This would indicate that the two main myosin II isoforms, IIA and IIB, play separate roles in the generation of cellular tension and cell-matrix interactions, with IIA perhaps playing a role in IIB localization/activation. There are many possibilities as to why the A and B isoforms of myosin II play different roles in cell rigidity and matrix interactions, however, a more in depth study into the regulation and activity of the isoforms during matrix rearrangement is needed to fully explain the mechanisms behind these actions.

Here we have shown that myosin II isoforms play separate and non-redundant roles in cell mediated matrix rearrangement. In addition, we have used a method of measuring ECM stiffness and elasticity that allows for a global and dynamic response from cells and matrices, as well as precise control over matrix components. These characteristics are known to have an effect on tumor development, therefore having a better understanding of how cancer cells rearrange and interact with the matrix and affect its rigidity *in vitro* may lead to innovations in diagnosis and treatment that could benefit public health. While we have described the differences in matrix interaction, further research into the mechanics of how myosin II isoforms are differentially involved in this interaction is needed.

## Supporting Information

S1 Fig Quantification of remaining myosin II isoforms in knockdown cells by immunofluorescent imaging. Cells were stained as outlined for actin and myosin II isoforms and imaged under low magnification. Levels of myosin II were quantified using Image J.
(TIF)
