# Supplementary figures and images for: Non-Muscle Myosin II Isoforms Have Different Functions in Matrix Rearrangement by MDA-MB-231 Cells
Source: PLoS One. 2015 Jul 2;10(7):e0131920. doi: 10.1371/journal.pone.0131920 (PMC4489869; doi:10.1371/journal.pone.0131920)

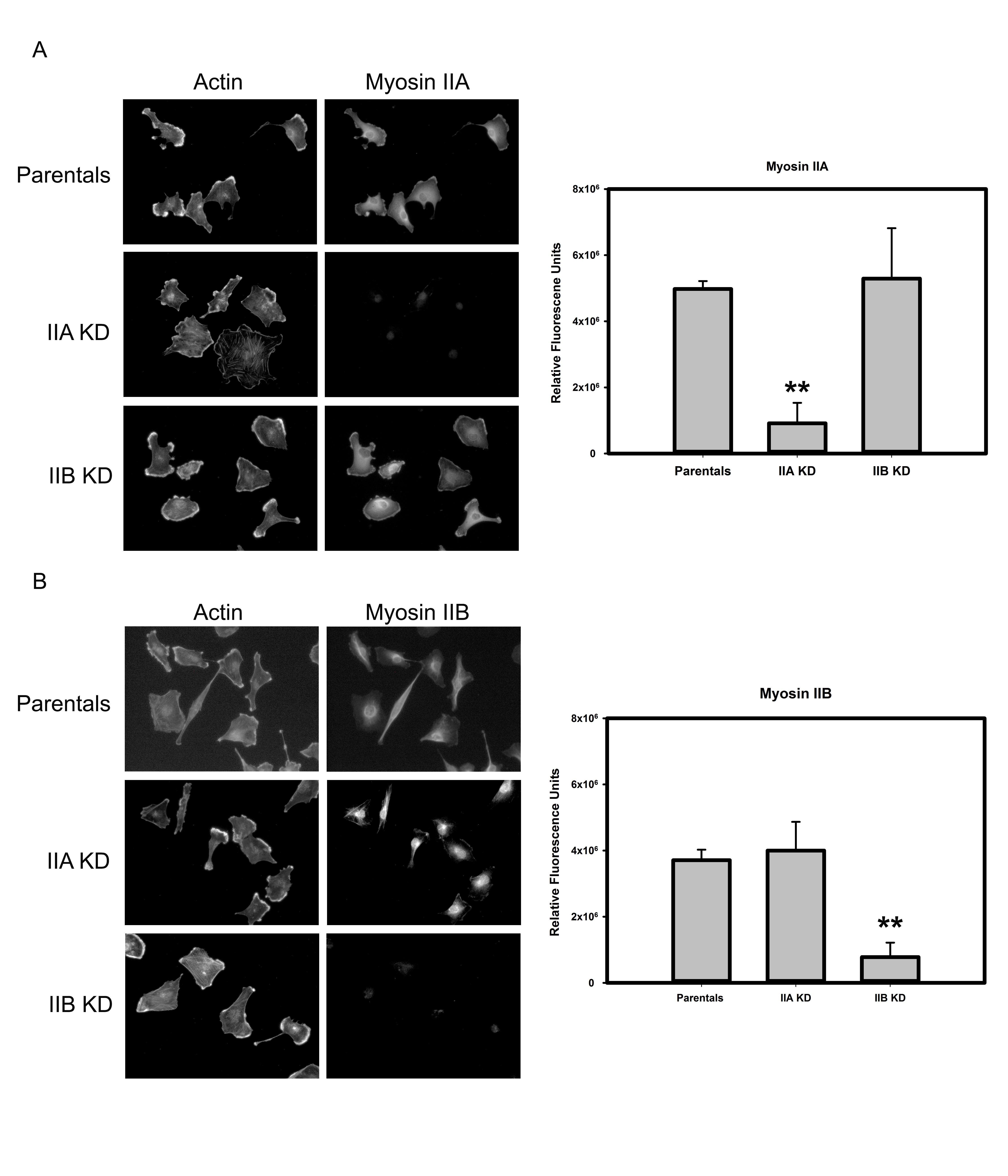

Supplement: S1 Fig — Cells were stained as outlined for actin and myosin II isoforms and imaged under low magnification. Levels of myosin II were quantified using Image J. (TIF) [file pone.0131920.s001.tif]
